# Knowledge, Attitude, and Practice Toward Prostate Cancer and Its Screening Methods Among Primary Care Patients in King Abdulaziz Medical City, Riyadh, Saudi Arabia

**DOI:** 10.7759/cureus.14689

**Published:** 2021-04-26

**Authors:** Ziyad F Musalli, Meshal M Alobaid, Abdulrahman M Aljahani, Meshari A Alqahtani, Sultan S Alshehri, Bader A Altulaihi

**Affiliations:** 1 Medicine, College of Medicine, King Saud Bin Abdulaziz University for Health Sciences, Riyadh, SAU; 2 Family and Community Medicine, King Abdulaziz Medical City, Riyadh, SAU

**Keywords:** prostate cancer, cancer screening, prostate cancer awareness, men’s health

## Abstract

Aim

The aim of the study was to evaluate the knowledge, attitude, and practice toward prostate cancer and its screening methods among patients attending primary care facilities in King Abdulaziz Medical City (KAMC) in Riyadh, Saudi Arabia.

Materials and methods

A cross-sectional survey was conducted on a random sample of 385 men. The questionnaire was distributed using a non-random sampling method (convenience sampling) that included 13 questions that assess the knowledge, attitude, and practice of adult male patients toward prostate cancer and its screening methods. The questions were divided into three general questions that test the knowledge, four questions that analyze patients’ opinions (attitude), and six questions on how patients are practicing screening methods with questions three and six being dependent on the answers to the questions that come before them.

Results

Around 64% of the participants had adequate knowledge about prostate cancer. Respondents with higher socioeconomic status demonstrated a higher level of knowledge about prostate cancer than the other groups. Regarding the attitude, more than 70% of the respondents believed that it is very important to screen for prostate cancer. About 23% of the participants had done some form of prostate screening test either prostate-specific antigen (PSA) or digital rectal exam (DRE); most of them were men older than 50 years.

Conclusions

The majority of the respondents to our survey demonstrated high general knowledge about prostate cancer. However, practice toward prostate screening methods was significantly low regardless of the positive attitude on the importance of screening. More data should be obtained to investigate the potential multifactorial reasons for such a low practice.

## Introduction

Prostate cancer is one of the most prevalent cancers around the world, which affects the prostate gland in males [1]. Prostate cancer is considered the fourth most common cancer globally and the second leading cause of cancer-related deaths in men [2]. According to the American Cancer Society, one in 41 American men will be diagnosed with prostate cancer [3,4]. Locally, according to the Saudi Cancer registry, 1739 cases were registered between January 2001 and December 2008. The highest incidence was in the eastern region, Riyadh, and Makkah [5]. All of the previous data present prostate cancer as the main health issue, both globally and locally.

One of the most important factors in preventing prostate cancer is regular screening and checkups. There are two types of screening that are commonly used in clinical practice, which are digital rectal exam (DRE) and prostate-specific antigen (PSA) [6]. DRE is highly recommended for men above the age of 40 years with previous family history or risk factors of prostate cancer and for men above the age of 50 years with no positive history [7]. On the other hand, PSA measurement is recommended for men between the age of 50 and 70 years [8]. Moreover, PSA is a very sensitive marker; however, its specificity is not very high in prostate cancer due to the false elevation in some benign prostatic diseases [9]. In the United States, screening has led to a down-staging of prostate cancer, with over 90% of men presenting with the localized and potentially curable disease at initial presentation.

Furthermore, several studies were conducted both globally and locally, which assessed the knowledge of people toward prostate cancer and its prevention methods. One study was done in Chile in which 377 men between the age of 50 and 90 years were surveyed regarding their knowledge of prostate cancer and screening practices. Results showed that 81% had some knowledge about prostate cancer, and 68% of those had done prostate screening [10]. Another study was done in Italy to analyze the knowledge, attitude, and practice toward prostate cancer and its prevention methods; 72.7% of the respondents have heard about PSA from their doctors, and 29% of those have done the PSA test [11].

Locally, a study was conducted in Jeddah, the largest city in the western province, to view prostate cancer screening awareness among men. This study had a sample size of 461 men over the age of 40 years. Among them, 352 (79%) of respondents had no knowledge regarding prostate screening methods. Surprisingly, only 10 people (2%) of the respondents had done prostate screening tests [12]. Another similar study was done in Riyadh comprising of 400 men, and only 10% of those had regular checkups [13]. All the studies conducted in Saudi Arabia showed a poor quality level of education and poor practice toward screening methods.

The current data demonstrate the level of awareness regarding prostate cancer screening in different areas of Saudi Arabia; however, there is a clear lack of literature regarding prostate screening in Riyadh, specifically, King Abdulaziz Medical City (KAMC). Thus, our research's main goal is to assess the knowledge, attitude, and practice regarding prostate cancer and its screening methods in three primary care centers governed by KAMC, Riyadh.

## Materials and methods

Study design, settings, and participants

This was a descriptive cross-sectional (questionnaire-based) study in Riyadh, Saudi Arabia, in which a group of male patients above the age of 40 years were questioned using a non-random sampling method (convenience sampling) about their knowledge, attitude, and practice toward prostate cancer and its screening methods. The study was conducted in three primary care centers (Khashm Alaan, Um Al Hamam, and Iskcon) that are governed by KAMC, Riyadh, Saudi Arabia. To calculate the sample size, the OpenEpi website was used. The population of patients attending primary care facilities in KAMC was found to be 240,000 divided into three primary care centers. They are distributed as 100,000 patients in Khashm Alaan center, 50,000 patients in Iskcon center, and 70,000 patients in Um Al Hammam center. Taking a confidence level of 95% as a convention and 5% as a margin of error, the sample size was found to be 385 with no specific percentage from each primary care center.

Data collection methods

Permission was taken from the director of each primary care center, and a questionnaire that was adapted, modified, and translated to Arabic by our team from a previous study was used [14]. The questionnaire includes 13 questions that assess the knowledge, attitude, and practice of male patients toward prostate cancer and its screening methods. The questions are divided into three general questions that test the knowledge, four questions that analyze the patients’ opinions (attitude), and six questions on how patients are practicing screening methods with questions three and six being dependent on the answers to the questions that come before them. After we obtained an informed consent from the participants, the questionnaires were distributed to male patients who meet the inclusion criteria for our study. The inclusion criteria were all male patients between 40 and 90 years old that visited one of the three primary care centers at least once throughout the last year so that they have active files. Lastly, any patient with severe comorbidities such as renal failure, heart failure, or his last visit to the hospital was more than one year ago or already suffering from any form of malignancy was excluded from the study.

Statistical analysis

The collected data from the questionnaire were analyzed using Statistical Package for Social Science, version 25 (IBM Corp., Armonk, NY, USA). The outcome variables will be scored as the sum of the related questions and then will be categorized based on the mean score into three levels as inadequate (1.00-1.66), moderate (1.67-2.33), and adequate (2.34-3.00) based on the average score. A score above the average will be considered adequate. The frequencies and percentages for categorical data such as education level, monthly income, and age were used to represent the data appropriately. Chi-square test and Likert scale analysis were used to measure and assess the rate of knowledge and analyze the attitude and practice toward prostate cancer and its screening methods among male patients. If the p-value is < 0.05, then there is a significant association when it comes to the variables, which in turn rejects the null hypothesis. Finally, the research project was ethically approved by the review board of King Abdullah International Medical Research Center (KAIMRC).

## Results

The study included a total of 385 males who were grouped based on their age, educational level, and monthly income. As per age, they were distributed into three categories: adults (40-49 years), (50-59 years), and elderly (above 60 years); most of them were between 40 and 49 years old (n = 179; 46.5%). In regard to the educational level, the participants were grouped into three categories: less than high school, high school, and college degree or higher. Most of the participants have a college degree or higher (n = 170; 44.2%). Also, as per the monthly income, the participants were divided into four categories as follows: less than 5000SR, 5000-10000SR, 10000-15000SR, and more than 15000SR. The majority of the respondents have a monthly income between 10000 and 15000SR (n = 121; 31.4%) (Table 1).

**Table 1:** Sociodemographic characteristics SR: Saudi Riyal.

Characteristics	n (%)
Age (n = 385)	
From 40 to 49 years	179 (46.5)
From 50 to 59 years	128 (33.2)
Above 60 years	78 (20.3)
Educational level (n = 385)	
Less than high school	91 (23.6)
High school	124 (32.2)
College or higher	170 (44.2)
Monthly income (n = 385)	
Less than 5000SR	60 (15.6)
5000-10000SR	108 (28.1)
10000-15000SR	121 (31.4)
More than 15000SR	96 (24.9)
Marital status (n = 183)	
Single	10 (5.5)
Married	158 (86.3)
Divorced	12 (6.6)
Widowed	3 (1.6)
All values are presented as numbers and percentages.	

Regarding the knowledge, about 64% of our sample size had adequate knowledge with a mean score of (1.92) (Table 2). The questionnaire showed that the younger age group (40-49 years old) had more knowledge about prostate cancer than other age groups; yet these differences were insignificant. In contrast, our data showed that participants who have college degree or higher had more knowledge about prostate cancer (n = 85; 50%). Moreover, respondents with a monthly income of more than 15000 SR had more knowledge than the others (n = 51; 53.1%). Thus, the association found between education and monthly income with patients’ level of knowledge was significant (Table 3).

**Table 2:** Patients' level of knowledge toward the prostate cancer

Statement	Mean	SD	Rank	Level of knowledge
Have you heard about Prostate Cancer?	2.58	0.813	1	Adequate knowledge
Do you know some kind of examination for cancer detection?	1.47	0.851	3	Inadequate knowledge
In your opinion, at what age should men be more concerned to take the examination?	1.7	1.007	2	Moderately adequate knowledge
Mean score	1.92			

**Table 3:** Testing the association between the sociodemographic characteristics with patients’ level of knowledge toward the prostate cancer (done by Chi-square test) SR: Saudi Riyal.

Demographics	Level of knowledge			P-value
	Inadequate knowledge n (%)	Moderately adequate knowledge n (%)	Adequate knowledge n (%)	
Age				
40–49 years	48 (26.8)	73 (40.8)	58 (32.4)	0.386
50–59 years	29 (22.7)	45 (35.2)	54 (42.2)	
More than 60 years	24 (30.8)	28 (35.9)	26 (33.3)	
Education				
Less than high school	34 (37.4)	33 (36.3)	24 (26.4)	0.000*
High school	37 (29.8)	58 (46.8)	29 (23.4)	
College or higher	30 (17.6)	55 (32.4)	85 (50.0)	
Monthly income				
Less than 5000SR	28 (46.7)	18 (30.0)	14 (23.3)	0.000*
5000-10000SR	30 (27.8)	47 (43.5)	31 (28.7)	
10000-15000SR	27 (22.3)	52 (43.0)	42 (34.7)	
More than 15000SR	16 (16.7)	29 (30.2)	51 (53.1)	
*Association found between education and monthly income with patients' level of knowledge at 0.05 level of significance.				

When we asked the participants about the importance of regular prostate cancer screening, the majority of the respondents had a good attitude regarding screening (71%) with a mean score of 2.13 (Table 4). Men above 60 years, (n = 53; 57.9%) displayed the best attitude. Only a minority of respondents had a poor attitude toward screening, which was most noticeable in men between 40 and 49 years old (n = 61; 34.1%). Lastly, no significance was noted between patient’s attitude and income/education (Table 5).

**Table 4:** Patients level of attitude regarding prostate examination

Statement	Mean	SD	Rank	Level of attitude
Is prostate examination the only way to diagnose prostate cancer?	1.83	1.403	3	Neutral
Is the adequate frequency of screening for men the same age of the interviewers annually?	2.05	1.11	2	Neutral
Should only those men with urinary symptoms screen?	1.74	1.201	4	Neutral
How important is it to perform prostate examination regularly?	2.91	0.35	1	Positive attitude

**Table 5:** Testing the association between the sociodemographic characteristics with patients’ level of attitude regarding prostate examination (done by Chi-square test) SR: Saudi Riyal.

Demographics	Level of attitude			P-value
	Negative attitude n (%)	Neutral n (%)	Positive attitude n (%)	
Age				
40–49 years	61 (34.1)	48 (26.8)	70 (39.1)	0.000*
50–59 years	31 (24.2)	34 (26.6)	63 (49.2)	
More than 60 years	9 (11.5)	16 (20.5)	53 (67.9)	
Education				
Less than high school	23 (25.3)	29 (31.9)	39 (42.9)	0.506
High school	32 (25.8)	32 (25.8)	60 (48.4)	
College or higher	46 (27.1)	37 (21.8)	87 (51.2)	
Monthly income				
Less than 5000SR	16 (26.7)	17 (28.3)	27 (45.0)	0.213
5000-10000SR	21 (19.4)	36 (33.3)	51 (47.2)	
10000-15000SR	34 (28.1)	24 (19.8)	63 (52.1)	
More than 15000SR	30 (31.3)	21 (21.9)	45 (46.9)	
*Association found between age and patients' level of attitude at 0.05 level of significance.				

Furthermore, the level of practice among our sample was found to be low with a mean score of 1.28, which represents 43% (Table 6). However, the only significant finding was the relationship between age and patients’ level of practice. The lowest level of practice among age groups was in men between 40 and 49 years old (n = 153; 58.5%). Moreover, the group with a higher level of practice was men between the age of 50 and 59 years (n = 22; 17.2%), which is still a very low number. When it comes to education and monthly income, the association between these demographic variables and the level of practice was insignificant (Table 7).

**Table 6:** Patients' level of practice regarding prostate examination PSA: Prostate-specific antigen.

Statement	Mean	SD	Rank	Level of practice
Has any physician advised you to screen for prostate cancer?	1.32	0.854	2	Low practice
Have you ever performed a prostate examination?	1.44	0.879	1	Low practice
Have you ever undergone a PSA?	1.06	0.713	3	Low practice
Mean score	1.28			

**Table 7:** Testing the association between the sociodemographic characteristics with patients’ level of practice regarding prostate examination (done by Chi-square test) SR: Saudi Riyal.

Demographics	Level of practice			P-value
	Low practice n (%)	Moderate practice n (%)	High practice n (%)	
Age				
40–49 years	153 (85.5)	16 (8.9)	10 (5.6)	0.000*
50–59 years	87 (68.0)	19 (14.8)	22 (17.2)	
More than 60 years	47 (60.3)	20 (25.6)	11 (14.1)	
Education				
Less than high school	68 (74.7)	14 (15.4)	9 (9.9)	0.552
High school	97 (78.2)	17 (13.7)	10 (8.1)	
College or higher	122 (71.8)	24 (14.1)	24 (14.1)	
Monthly income				
Less than 5000SR	50 (83.3)	8 (13.3)	2 (3.3)	0.125
5000-10000SR	75 (69.4)	21 (19.4)	12 (11.1)	
10000-15000SR	93 (76.9)	15 (12.4)	13 (10.7)	
More than 15000SR	69 (71.9)	11 (11.5)	16 (16.7)	
*Association found between age and the patients' level of practice at 0.05 level of significance.				

## Discussion

Our study presents an overview of how prostate cancer screening is perceived among a sample of the Saudi population. Although prostate cancer screening, specifically PSA test, has received negative remarks in recent years, it remains the gold standard test for screening [15]. The controversy surrounding prostate screening has been a major topic in the field of urology and family medicine. However, due to the failure of primary prevention methods and the use of pharmacological treatment to lower the incidence of the disease, secondary prevention should not be discouraged, especially if done by a skilled physician that customized the necessity of the ordered tests according to the patient’s modifiable and non-modifiable risk factors to prevent overdiagnosis and overtreatment. Furthermore, decisions regarding prostate screening must be based on the wish of an informed patient under the appropriate clinical setting [16].

The majority of men in our sample had adequate general knowledge regarding prostate cancer (64%). Similar values have been reported in other studies conducted in Italy and Jamaica [11,17], with 82% and 96%, respectively. In comparison, this value was higher than some data reported from different countries. For example, a study done in South Africa found that only 45.7% of men attending urologic outpatient clinics had adequate knowledge [18]. Moreover, socioeconomic status played an important role in our study in which men with college degree or higher and those with a monthly income higher than 15000SR had better knowledge regarding prostate cancer screening. These findings could be attributed to the fact that those with a college degree or higher might have easier access to sources that constantly emphasize the significance of prostate cancer screening such as social media and online scientific articles.

Regarding knowledge of prostate screening methods, primarily PSA test, we found that 41.8% of men had heard of it before. This value is lower than the 72.7% reported in a survey conducted in Italy [11]. The level of education played a huge role in our data. Most men who heard about prostate screening methods received higher levels of education (college or higher).

In addition, most of the participants in our survey had a good attitude toward prostate screening with 71% claiming that it is “very important” to screen for prostate cancer. This adequate number did not seem to have large repercussions on the practice of those patients. Thus, the level of practice among our sample was found to be low with a percentage of 43%. Moreover, 9.6% of the respondents have undergone a PSA test. This finding was similar to a previous study conducted in Riyadh, Saudi Arabia, in which only 10% had done PSA test [13]. Globally, higher results were found in Italy (29.6%) and South Africa (28.3%) [11,18]. Furthermore, there is a good chance that the percentage of patients who did the PSA test is not a good representative for the practice in our sample size due to the fact that 23% of respondents have done some type of prostate screening either DRE or PSA. Thus, doctors’ medical judgments on when PSA is indicated for patients with normal DRE could have been the reason behind this low percentage. The issue lies in whether those patients in our sample had undergone prostate screening for checkup purposes or due to the presence of symptoms. Around 45% of those who did the tests, patients above the age of 50 years had urological symptoms that led them to seek medical attention; therefore, it was not technically for screening purposes.

Such low figures of prostate cancer screening, which was found in our study could be multifactorial. The most important one, however, is the lack of physicians’ advice and guidance when it comes to the importance of screening. Only 19.2% of our sample claimed that a physician discussed their options regarding prostate screening and its significance. This was similar to national and global studies, which demonstrated that the main reason given for not attending screening services was the lack of doctors' input on the matter [19,20]. Another crucial reason that could explain these figures was the poor attitude given by some of our survey respondents when it comes to the timing of screening. Even though most of the respondents had a good attitude toward the importance of prostate cancer screening, around 37% of them thought that only those with urological symptoms should screen for prostate cancer. This type of attitude points toward a fundamental misunderstanding of the concept of screening and should be addressed in future local campaigns and by physicians as well.

There were some potential limitations encountered in this study. First, a questionnaire-based cross-sectional method was used, so there was no apparent association between dependent and independent variables. Second, our survey was self-administered, and it might have been possible for respondents to describe their perceived understanding of the healthy behavior and not their own real behavior. Lastly, some participants filled the questionnaire without direct supervision of a research member. Thus, it is possible that some of them sought for information before answering the survey.

## Conclusions

In conclusion, although this study showed an acceptable level of knowledge regarding prostate cancer and its screening methods, the practice within our sample size was poor. Therefore, more data should be obtained on finding the multifactorial reasons for such behavior. Also, the lack of physicians’ advice was a huge factor in our study; therefore, maybe future research targeting family physicians and their attitude toward prostate screening could be beneficial in helping to understand their ideas and beliefs when it comes to screening. Lastly, local campaign organizers and community doctors are encouraged to raise awareness when it comes to the significance of screening as it is an important tool for prevention due to the failure of primary methods in lowering the incidence of the disease.

## Appendices

**Table 8:** Knowledge, attitude, and practice (percentage by each question in the survey)

Question	n (%)
Have you heard about Prostate Cancer? (n = 385)	
Yes	305 (79.2)
No	80 (20.8)
If yes where/who mentioned it? (n = 305)	
TV/Radio/Newspaper	134 (43.9)
Friends	17 (5.6)
PSF (family health program)	25 (8.2)
Other health service	58 (19.0)
Relatives	70 (23.0)
Other	1 (0.3)
Do you know some kind of examination for cancer detection? (n = 385)	
Yes	91 (23.6)
No	294 (76.4)
If yes, types of examination you know (n = 91)	
Rectal exam	53 (58.2)
PSA blood test	13 (14.3)
Rectal exam/PSA blood test	25 (27.5)
In your opinion, at what age should men be more concerned to take the examination? (n = 385)	
30–<40 years old	72 (18.7)
40–50 years old	159 (41.3)
>50 years old	88 (22.9)
Does not know	66 (17.1)

Has any physician advised you to screen for prostate cancer? (n = 385)	
Yes	74 (19.2)
No	287 (74.5)
Does not know/does not remember	24 (6.2)
Have you ever performed a prostate examination? (n = 385)	
Yes	90 (23.4)
No	284 (73.8)
Does not know/does not remember	11 (2.9)
Reason for request of prostate examination (n = 90)	
Presented symptoms	42 (46.7)
Cancer case in the family	6 (6.7)
Prevention	36 (40.0)
The participant requested the examination	6 (6.7)
When was the last time you underwent the examination? (n = 90)	
Over three years ago	21 (23.3)
Between one and two years	23 (25.6)
Less than one year ago	46 (51.1)
Have you ever undergone a PSA? (n = 385)	
Yes	37 (9.6)
No	299 (77.7)
Does not know/Does not remember	49 (12.7)
When was the last time you underwent a PSA? (n = 37)	
Over three years ago	6 (16.2)
Between one and two years	7 (18.9)
Less than one year ago	24 (64.9)

Is prostate examination the only way to diagnose prostate cancer? (n = 385)	
Yes	224 (58.2)
No	34 (8.8)
Does not know	127 (33.0)
Is the adequate frequency of screening for men the same age of the interviewers annually? (n = 385)	
Does not know	50 (13.0)
Only when there are symptoms	77 (20.0)
Every three to five years	61 (15.8)
Every two years	197 (51.2)
Should only those men with urinary symptoms screen? (n = 385)	
Yes	145 (37.7)
No	175 (45.5)
Does not know	65 (16.9)
How important is it to perform prostate examination regularly? (n = 385)	
Doesn't matter	8 (2.1)
Little or not important at all	18 (4.7)
Important	359 (93.2)
All values are presented as number and percentage	
